# *Arabidopsis GAAP1* to *GAAP3* Play Redundant Role in Cell Death Inhibition by Suppressing the Upregulation of Salicylic Acid Pathway Under Endoplasmic Reticulum Stress

**DOI:** 10.3389/fpls.2019.01032

**Published:** 2019-08-27

**Authors:** Wei Wang, Xin Li, Manli Zhu, Xiaohan Tang, Zhiying Wang, Kun Guo, Yan Zhou, Yue Sun, Wei Zhang, Xiaofang Li

**Affiliations:** School of Life Sciences, East China Normal University, Shanghai, China

**Keywords:** *Arabidopsis thaliana*, *GAAP*, endoplasmic reticulum stress, cell death, unfolded-protein response, salicylic acid

## Abstract

The unfolded protein response (UPR) is activated to sustain cell survival by reducing misfolded protein accumulation in the endoplasmic reticulum (ER). The UPR also promotes cell death when the ER stress is severe. However, the underlying molecular mechanisms of UPR activity regulation and cell death transition are less understood in plants. *Arabidopsis GAAP1* and *GAAP3* are involved in the regulation of UPR and cell death. Five *GAAP* gene members are found in *Arabidopsis*. Here, we analyzed the function of *GAAP2* in addition to *GAAP1* and *GAAP3* in ER stress response using single, double, and triple mutants. Results showed that single or double or triple mutants reduced plant survival and enhanced cell death under ER stress. And the sensitivity increased with the number of mutation genes increase. Quantitative real-time polymerase chain reaction analysis showed that mutation in triple genes promoted UPR signaling when confronted with mild ER stress, advanced SA target genes upregulation when confronted with severe stress. Moreover, Quantitative detection by UPLC-ESI-MS/MS showed that ER stress upregulated salicylic acid (SA) content in plants. These data suggest that *GAAP1* to *GAAP3* played redundant roles in cell death resistance and fine tuning UPR activation. And the anti-cell death function of *GAAP*s might be achieved by impairing the up-regulation of the SA pathway under ER stress.

## Introduction

Plants as sessile beings have evolved sensitive detection systems and sophisticated signal transduction mechanisms that help them cope under numerous stress conditions. The endoplasmic reticulum (ER) is a central organelle for protein folding and maturation. Under various adverse conditions, protein folding process will be affected resulting in the accumulation unfolded or misfolded proteins in the ER, which named as ER stress. The unfolded protein response (UPR) is a cellular response that is highly conserved in eukaryotes to obviate ER stress (Liu et al., 2012). In plants, at least two UPR pathways have been identified. One UPR pathway involves the proteolytic cleavage and release of different bZIP transcription factors (TFs), such as bZIP28/17 from the ER membrane. The displaced TFs enter the nuclei and activate the transcription of specific genes involved in various ER quality-control processes under ER stress DDIN EN.CITE (Liu and Howell, 2010a; Walter and Ron, 2011). Another pathway is sensed by the molecule IRE1. Activated IRE1 catalyzes the splicing messenger RNA encoding transcription factor bZIP60 to up-regulate the UPR-related genes encoding factors that aid in protein folding and degradation. And IRE1 also degrades mRNAs encoding proteins in the secretory pathway referred to as regulated IRE1-dependent decay (RIDD) to reduce the amount of protein entering the ER in UPR. In addition, activated IRE1 can induce autophagy to mitigate ER stress TA (Deng et al., 2011; Nagashima et al., 2011; Humbert et al., 2012; Brewer, 2014). When cytoprotective outcomes are insufficient for ER homeostasis restoration under severe or chronic stress, the UPR triggers cell death program (Liu et al., 2007; Liu and Howell, 2010b; Yang et al., 2014). However, minimal information is known about the regulation of ER-stress sensor activities and about the determination of different outcomes between cell survival and death effects in plants. Under ER stress, IRE1 and bZIP28 can induce PCD by up-regulating *NAC089*, which promotes cell death by activating the expression of downstream genes, such as *NAC094* and *ATPase* (Yang et al., 2014). In addition, the cell death domain (DCD)-containing asparagine-rich proteins (NRPs) have been identified in plant as transducers of a cell death signal derived from various stresses. In *Arabidopsis*, DCD/NRP-mediated signaling induces cell death by sequentially activating *AtNRPs*, *ANAC036* and *gVPE* genes under osmotic or ER stress (Reis et al., 2016).

The UPR plays a fundamental role in plant immunity and abiotic stress responses (Moreno et al., 2012). Under biotic stress, plants trigger a robust disease resistance at the site of infection. Stimulation of defense responses occurs not only locally but also throughout the plant, known as systemic acquired resistance (SAR). Activation of the SAR pathway involves an increase in the cellular concentration of the immune signal salicylic acid (SA). SA induces pathogenesis-related (PR) genes and a large set of ER-resident genes to ensure proper folding and secretion of the PR proteins .CITE.DATA (Wang et al., 2005; Nishimura and Dangl, 2010). SA pathway also plays a key regulatory role in the resistance and promotion of PCD under stress (Yun and Chen, 2011; Gao et al., 2017). Exogenous SA has been reported to activate two plant UPR pathway genes (Nagashima et al., 2014). However, it is not clear whether cell death induced under ER stress is relevant to SA pathway. We have previously reported that *Arabidopsis GAAP1* and *GAAP3*, a sub-family of BI-1 factors, are involved in UPR and PCD regulation. *GAAP1*/*GAAP3* inhibits ER stress-induced cell death and promotes plant-growth recovery by attenuating the IRE1-mediated UPR process after the ER stress relief (Guo et al., 2018). Five *GAAP* gene members are found in *Arabidopsis*. Do the homologous members show similar function? How is the function of *GAAPs* in anti-cell death achieved? *GAAP2*, also named as *BIL4*, localizes to the *trans*-Golgi network/early endosome, late endosome, and vacuolar membranes. *GAAP2*/*BIL4* regulates cell elongation and brassinosteroid (BR) signaling *via* the regulation of BRI1 localization. (Yamagami et al., 2017). We analyzed the function of *GAAP2* in addition to *GAAP1* and *GAAP3* in ER stress. The analysis of double and triple mutants showed that *GAAP1* to *GAAP3* played redundant roles in inhibiting cell death and delaying the ER stress-induced UPR activation. Quantitative detection by UPLC-ESI-MS/MS showed that ER stress upregulated SA content in plants. The quantitative real-time reverse-transcription polymerase chain reaction assay showed that the anti-cell death function of *GAAP*s might be achieved by impairing the up-regulation of the SA pathway under ER stress.

## Materials and Methods

### Plant Material and Growth Conditions

*A. thaliana* of ecotype Columbia-0 (*Col*) plants and T-DNA insertion mutants in the Col-0 background were used. The mutants *gaap2-1* (CS814747) and *gaap2-2* (SALK_052507C) were isolated from the Salk T-DNA collection.

*gaap1-1*, *gaap3*, and *gaap1gaap3* have been previously mentioned (Guo et al., 2018). The T-DNA insertion site was confirmed by plant genomic DNA polymerase chain reaction (PCR) amplification with T-DNA and gene-specific primers. The double and triple mutants *gaap1gaap2, gaap2gaap3*, and *gaap1gaap2gaap3* were generated by hybridization. *gaap1-1* and *gaap2-1* were used in the double and triple mutants. Overexpression lines 35S::*GAAP1*, 35S::*GAAP3*, and 35S::*GAAP3*-FLAG have been previously reported (Guo et al., 2018).

Methods for plant growth and the ER stress seedling sensitivity have been previously described (Guo et al., 2018). Briefly, tunicamycin (TM), an inhibitor of N-linked glycosylation, and dithiothreitol (DTT), a redox reagent, were used to induce ER stress. Sterile seeds were cultured on medium containing low-concentration TM for various times. Alternatively, unless otherwise specified, 3-day-old seedlings grown on a 1/2 MS agar plate were transferred onto a plate containing different concentrations of TM or DTT for various times. To test growth recovery, 4-day-old seedlings that had been infiltrated with 1/2 MS liquid salt containing 0, 0.15, and 1.0 μg ml^−1^ TM for 6 h were transferred onto 1/2 MS solid medium. The fresh weight of seedlings was determined during recovery time.

For quantitative real-time reverse-transcription PCR (qPCR) assay, unless specifically noted, 7-day-old seedlings were incubated with 1/2 MS liquid medium containing different concentrations of TM for various times as indicated. To analyze the effect of various abiotic stresses on the expression of *GAAP2* gene, the 7-day-old seedlings were incubated with liquid medium containing 0.5 μg ml^−1^ TM, 50 mmol L^−1^ ABA, or transferred to light incubator with 37°C (HT) or 15°C (LT) for 6 h, respectively. To analyze the induction of UPR marker genes under mild ER stress, seedlings were treated with 80 ng ml^−1^ TM for 6 h. Seedlings were treated with 2.0 or 5 μg ml^−1^ TM for 6 h, which were defined as acute ER stress condition.

All the data in the paper were from at least three independent experiments subjected to Student’s t-test or two-way ANOVA analysis. An α level of 0.05 was used for statistical significance.

### Plasmid Construction

To generate promoter-GUS (β-d-glucuronidase) constructs, we cloned genomic DNA sequence corresponding to 903 bp upstream of the ATG codon of the *GAAP2* ORF into pBI101.1 vector. A DNA sequence of 1,925 bp in length, including the promoter and the *GAAP2* gene, was cloned into pBI101.1 vector to produce the p*GAAP2*::*GAAP2*. The primer pairs are listed in **Supplementary Table S1**, and all generated constructs were confirmed by sequencing.

### Plant Transformation and Transgenic Plant Analysis

*Agrobacterium tumefaciens* strain GV3101 was used. Transformation was performed using the floral-dip method (Clough and Bent, 1998). The phenotypic effects of p*GAAP2*::*GAAP2* in transgenic *gaap2-1* plants were analyzed in more than four independent transgenic lines. Transgenic plants carrying *GAAP2*::GUS in Col were further selected for GUS activity detection as previously described (Li et al., 2009; Guo et al., 2018).

### Histochemistry and Microscopy

The 3-day-old seedlings that were vertically cultured were incubated with 1/2 MS liquid medium containing different concentrations of TM for various times as indicated. Propidium iodide (PI) staining as fluorescent indicator of cell-membrane permeability and ion leakage assay were performed as previously described (Watanabe and Lam, 2008; Duan et al., 2010). Briefly, the seedlings were immersed in the 1/2 MS liquid salt solution containing 100 μg ml^−1^ PI (Sigma) and incubated for 2 min on ice under dark. And then the seedlings were washed with 1/2 MS liquid salt several times. The pictures of staining roots were then taken under fluorescence microscope (Olympus BX53, excitation = 535 nm; emission = 615 nm) with exposure time 40 ms. The fluorescence intensity of the root was measured with Image J. Cell death detection by trypan blue staining was performed as previously described (Ning et al., 2002; Duan et al., 2010). Four to five biological replicates were conducted for each staining, and at least 20 samples were determined for each genotype every replicate.

### Quantitative Real-Time Reverse-Transcription PCR (qPCR)

Total RNA from different tissues was extracted from a frozen tissue using TRIZOL reagent (Invitrogen), and the first cDNA strand was generated in accordance with the instructions of Superscript RT (Toyobo, Japan). qPCR analysis was performed using three to six independent biological replicates, and data were analyzed as previously reported (Schmittgen and Livak, 2008; Li et al., 2013). The relative expression levels of UPR, cell death and salicylic acid (SA) target genes were the level of each gene in different plant genotypes normalized to the level in the wild-type control, both of which were normalized to *ACTIN8* expression. The specific primers for each gene were described previously (Guo et al., 2018) or listed in **Table S1**. Two-way ANOVA was performed, and Tukey’s range (honest significant difference) test was used to determine significant differences among genotypes or different treatments.

### Salicylic Acid Measurement

Quantitative detection of SA content in samples was conducted by UPLC-ESI-MS/MS analysis. The mass spectrometry system uses the API4500 triple quadrupole tandem mass spectrometry detection system of AB Sciex Corporation of the United States. The determination of SA content was assisted by Shanghai Luming Biotechnology Co., Ltd. Total SA were extracted and measured from 3-week-old plants grown at 22°C or sprayed with 0.3 μg ml^−1^ TM for 48 h. To extract SA, the appropriate amount of lyophilized samples were put in 1.5 ml extraction liquid (methanol: water: formic acid = 7.9:2:0.1) with steel balls, and were crushed for 2 min followed by ultrasonication for 30 min at low temperature and then incubated overnight at 4°C. The supernatant was removed by centrifugation at 13,000 rpm for 1 min at 10°C, and the filter residue was added by 1.0 ml extraction liquid followed by ultrasonication for 30 min and further immersed for 60 min at 4°C. The supernatant by centrifugation was combined with the extraction at the first time and the methanol was evaporated. Then the extracted residue was purified by MAX solid phase extraction column. The eluate was concentrated and dried in vacuo. The last extracted residue was dissolved in 100 μl methanol and filtered with a 0.2 μm nylon membrane before detection. And the data was obtained from at least four biological replicates.

## Result

### *GAAP2* Preferentially Expressed in Young Tissues and the Reproductive Organs

**Figure 1:**
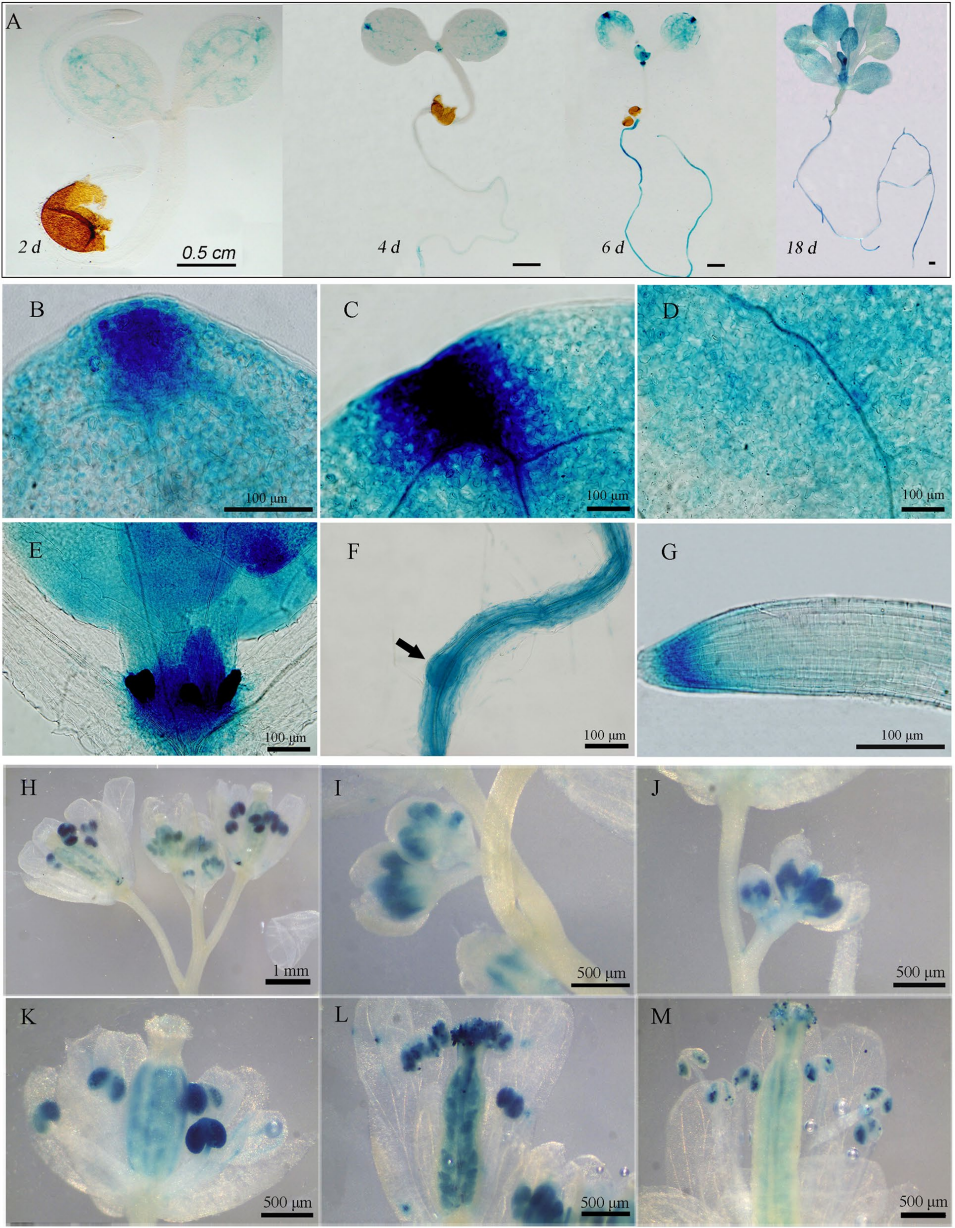
Expression pattern of *GAAP2* assayed by *GAAP2* promoter fusion GUS reporter assay. **(A–G)** The expression pattern of *GAAP2* during the vegetative growth stage. The GUS staining of *GAAP2* in the whole seedling at 2 days to 18 days **(A)**, euphylla tip **(B)**, cotyledon tip **(C)**, cotyledon veins **(D)**, leaf primordium and shoot apical **(E)**, lateral root primordia, **(F)** and primary root tips **(G)** of 6 day-old *pGAAP2::GUS* transgenic plants. The expression pattern of *GAAP2* during the reproductive growth stage **(H–M)**. The GUS staining of *GAAP2* in the whole inflorescence **(H)**, flower buds at stages 9–11 **(I** and **J)**, 12 **(K)**, 14 **(L)**, and 15 **(M)**.

**Figure 2:**
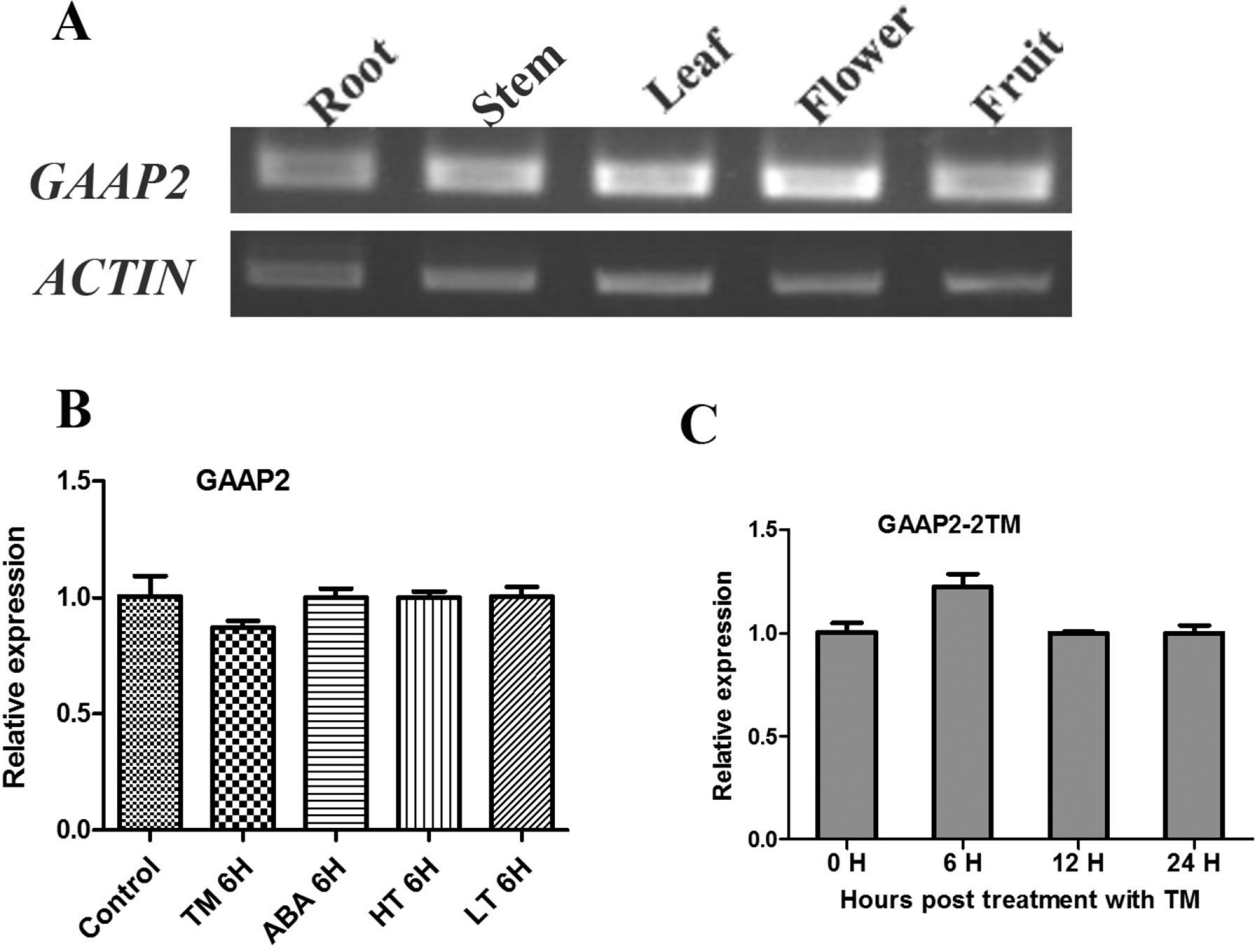
Transcript level of GAAP2 was not affected by various abiotic stresses. **(A)** The transcription level of *GAAP2* in different tissues of 50-day-old plants assayed by RT-PCR. **(B–C)** The transcription level of *GAAP2* in 7-day-old seedlings treated with different stresses assayed by qRT-PCR. The levels in seedlings in the absence or presence of 0.5 μg ml^−1^ TM, 50 mmol L^−1^ ABA, 37°C (HT) and 15°C (LT) for 6 h, respectively **(B)**. The levels in seedlings in the presence of 2 μg ml^−1^ TM at different hours **(C)**. The relative gene expression was normalized to the level of the control and was normalized to *ACTIN8* expression. Data are from three biological replicates (± SD). Statistical significance was analyzed in accordance with Tukey’s range (honest significant difference) test and two-way ANOVA (*p*< 0.05).

To illustrate the expression patterns of *GAAP2*, we generated three lines of *GAAP2* promoter fusion GUS (p*GAAP2*::GUS) expression transformants and performed GUS staining analysis in addition to RT-PCR assay. *GAAP2* was expressed in various tissues, including cotyledon, leaf, root and reproductive organs (**Figures 1** and **2A**). During vegetative development, strong expression signals were found in cotyledon tip, leaf primordia, root apical, and lateral root primordia, followed by the leaf vein (**Figures 1A–G**). With flower development, GUS activity was strong in the anther and ovules but not in the petals (**Figures 1H–M**).

The transcription of *GAAP1* and *GAAP3* genes are induced by ER stress and abiotic stress (Guo et al., 2018). The effects of tunicamycin (TM) or ABA, high or low temperature treatment on the transcription of *GAAP2* gene during the seedling stage were tested. As shown in **Figures 2B, C**, *GAAP2* expression was unchanged by the abiotic stresses.

### Mutations of *GAAP2* Enhanced the Plant Sensitivity to ER Stress

**Figure 3:**
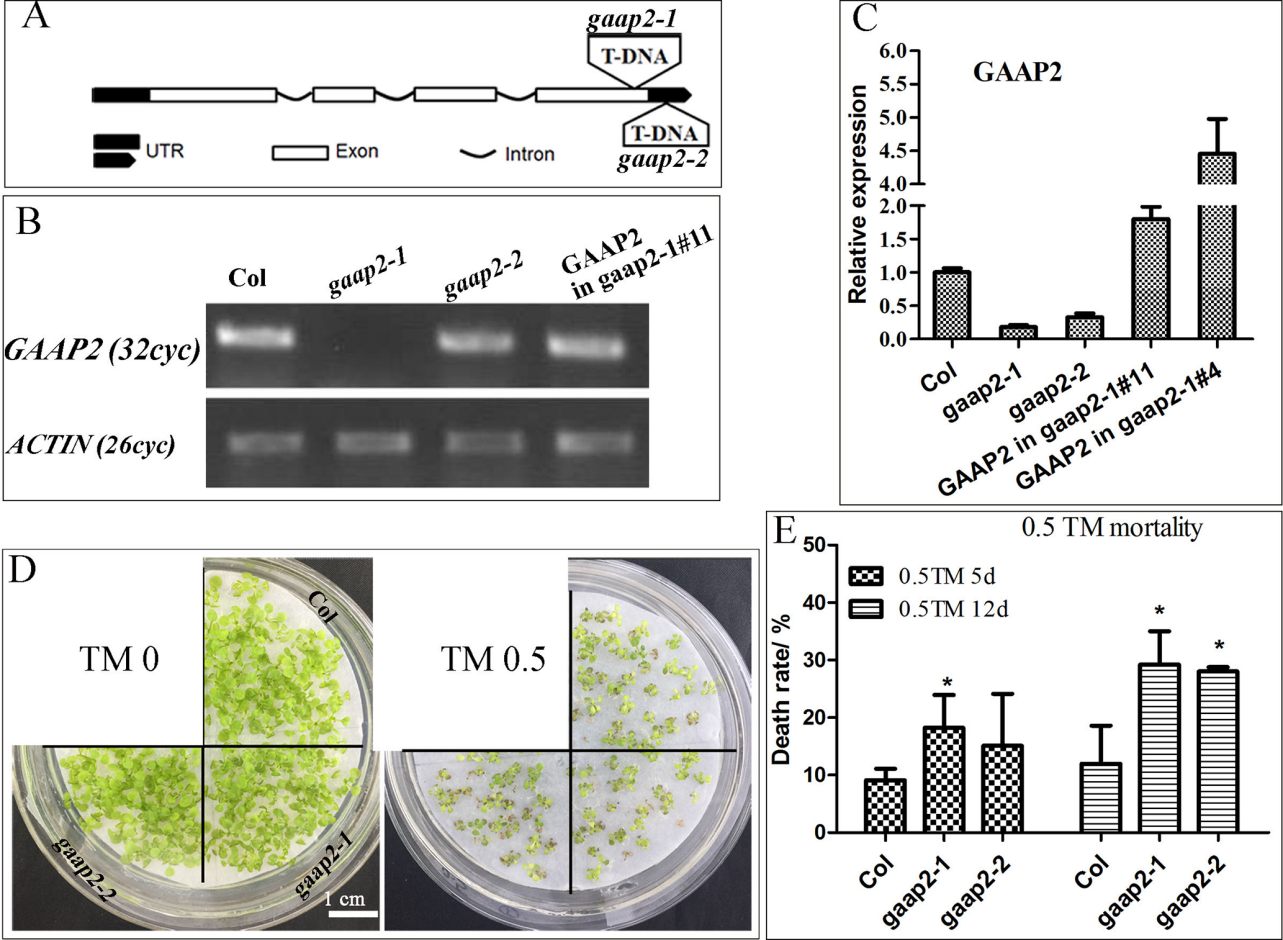
*GAAP2* mutation increased the sensitivity of seedlings to TM treatment. **(A)** The schematic of T-DNA insertion sites in *gaap2-1* and *gaap2-2*. **(B–C)** The expression of *GAAP2* in 7-day-old seedlings of Col, *gaap2-1*, *gaap2-2*, and two transgenic lines with GAAP2 in *gaap2-1*was assayed by RT-PCR **(B)** and qRT-PCR **(C)**. **(D–E)** GAAP2 mutation enhanced TM-induced plant death. Col, *gaap2-1* and *gaap2-2* seedlings were grown on 1/2 MS medium for 3 days and then transferred to 1/2 MS medium supplied with 0 or 0.50 μg ml^−1^ TM for another 12 days **(D)**. The death rates of seedlings treated with 0.5 μg ml^−1^ TM for 5 and 12 days **(E)**. Error bars depict a standard error of approximately three to four independent experiments. Significant differences compared with Col plants, as indicated by asterisks (χ*^2^* test, *p < 0.05, n > 80).

**Figure 4:**
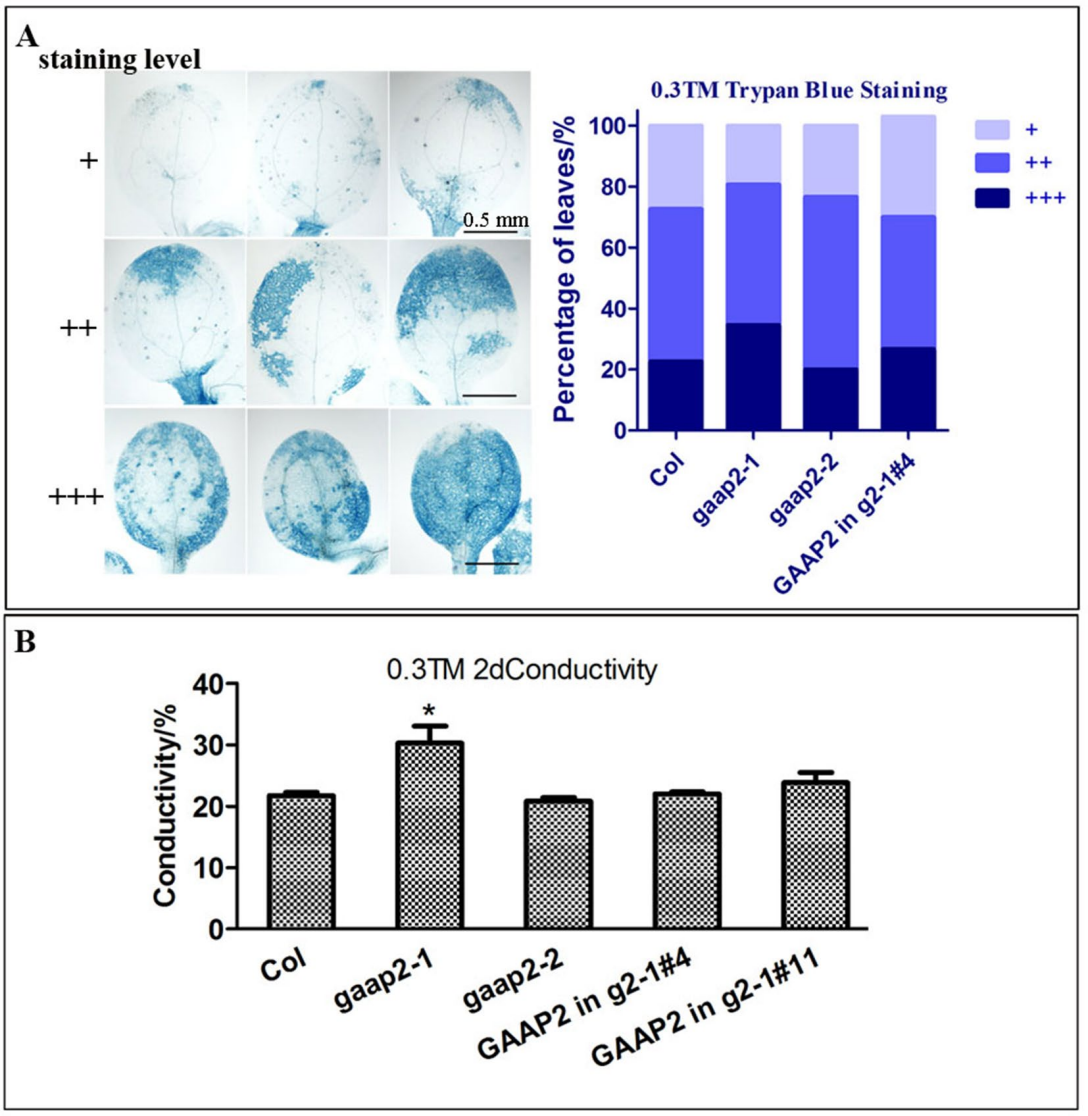
*GAAP2* mutation enhanced cell death induced by ER stress. **(A)** The seedlings of 3-day-old Col, *gaap2-1*, *gaap2-2* and *GAAP2 in gaap2-1*, which were soaked in 0.3 μg ml^−1^ TM for 48 h, were stained with trypan blue. The staining intensity of the cotyledons was classified in three levels, and cell death severity was analyzed by quantifying the staining degree. Data are shown as mean ± SD (*n* >25). **(B)** Shoots were collected from the samples obtained from the different sets of seedlings performed for **(A)** and then subjected to ion leakage measurements. Data are expressed as mean ± SD. Significant differences compared with Col plants are indicated by asterisks (*p < 0.05, n = 5).

To determine whether *GAAP2* exhibits similar function as *GAAP1* and *GAAP3* in ER stress resistance, we obtained loss-of-function mutant *gaap2-1* and gene knock-down mutant *gaap2-2* in addition to transgenic line expressing *GAAP2* driven by its own promoter in *gaap2-1* (*GAAP2* in *gaap2-1*) (**Figures 3A–C**). All mutants and transgenic plants did not exhibit evident aboveground growth defects under normal growth condition. When seedlings were transferred to a medium containing TM (0.5 μg ml^−1^) to grow for a couple of days, plant survival decreased with increasing time. The mortality rate was highest in *gaap2-1*, followed by *gaap2-2* (**Figures 3D, E**), which were consistent with the *GAAP2* gene level in the plant. The effect of *GAAP2* on ER stress resistance at the cellular level was further analyzed. When seedlings of *gaap2* mutants and complement lines was treated with 0.3 μg ml^−1^ TM for 48 h, cell death and membrane permeability were determined by trypan blue staining and electrical conductivity, respectively. The cells of *gaap2-1* mutant showed the most damage, whereas *gaap2-2* and *GAAP2* in *gaap2-1* showed similar cell damage as Col (**Figure 4**). These data suggested that the lost *GAAP2* enhanced the cell death under ER stress induced by TM and *GAAP2* conferred increased ER stress tolerance.

When seedlings cultured in sterile medium or growing on soil were treated with DTT, the death rate and leaf etiolation rates were significantly higher in both *gaap2* mutants than that in Col, *GAAP2* in the mutant background partially restored the mortality rate and etiolation (**Supplementary Figure S1**).

### Plant Sensitivity Toward ER Stress Increased With the Increasing Mutant Gene Number of *GAAP1*, *GAAP2*, and *GAAP3*

**Figure 5:**
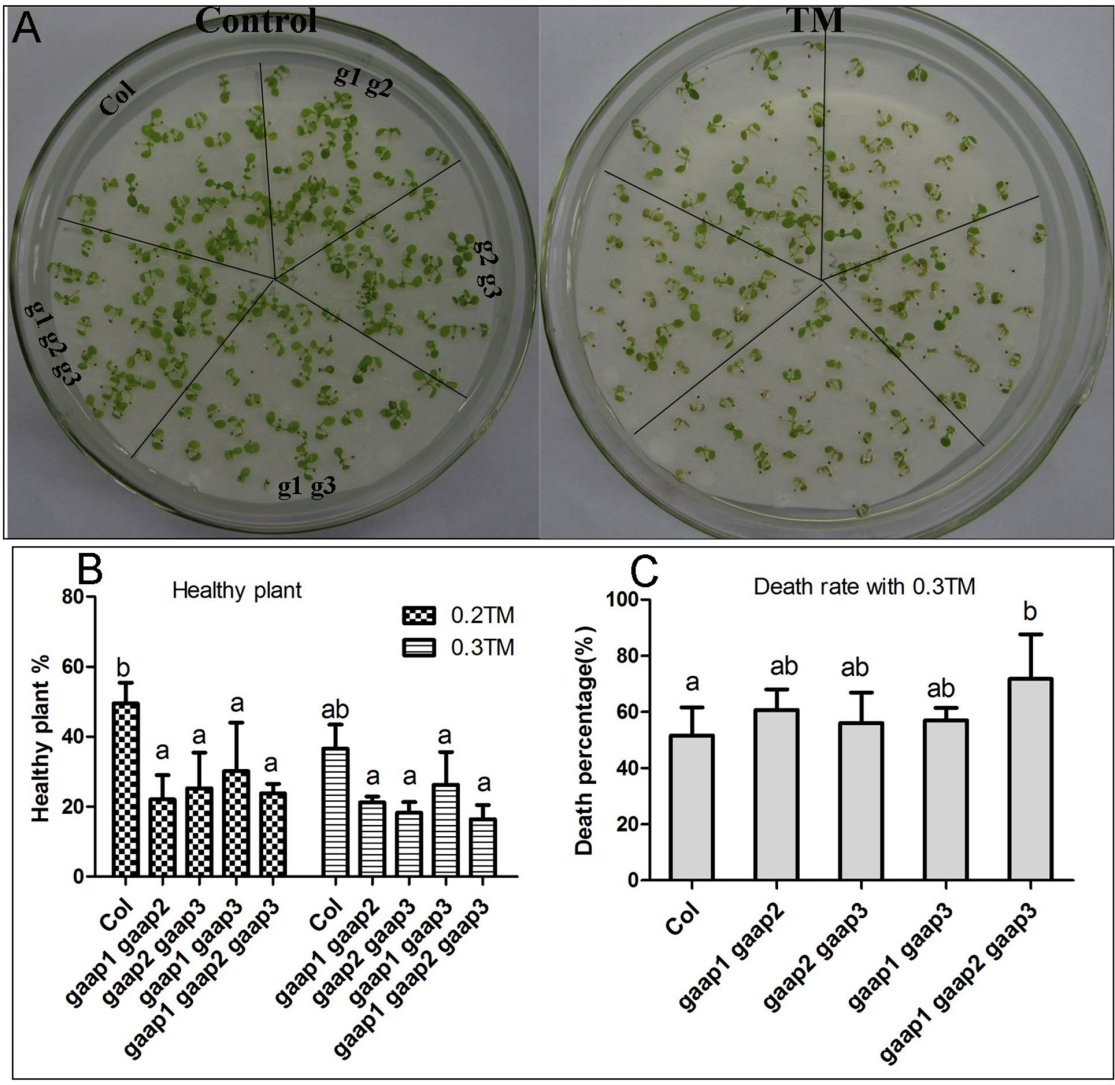
Plant sensitivity toward TM treatment increased with the increasing mutant gene number of *GAAP1*, *GAAP2* and *GAAP3*. **(A)** The growth of Col, *gaap1gaap2*, *gaap2gaap3*,* gaap1gaap3* and *gaap1gaap2gaap3* seedlings on 1/2 MS medium for 3 days and then transferred on 1/2 MS medium supplied with 0–0.20 μg ml^−1^ TM for another 6 d. **(B–C)**. Healthy plant **(B)** and death rates **(C)** of seedlings, which were treated with 0.2 and 0.3 μg ml^−1^ TM for 6 days. Error bars depict the standard error of four independent experiments, and different letters indicate significant differences between different plants subjected to χ test (*p < 0.05, n > 100).

*GAAP1* and *GAAP3* display redundant function in plants resistance to ER stress (Guo et al., 2018). To determine if *GAAP1*, *GAAP2*, and *GAAP3* play functional redundant role in enhancing plant resistance to ER stress, we generated double mutants, *gaap1gaap2*, *gaap2gaap3*, and triple mutant *gaap1gaap2gaap3* (**Supplementary Figure S2**). No obvious defects were found in double and triple mutants except for their slightly early bolting time. In terms of health rate, mortality and germination rate, both the seedling damage and growth inhibition by TM treatment were most severe in triple mutant, followed by double mutants (**Figure 5** and **Supplementary Figure S3**), which conferred the function redundancy of the three *GAAP* homologous genes.

**Figure 6:**
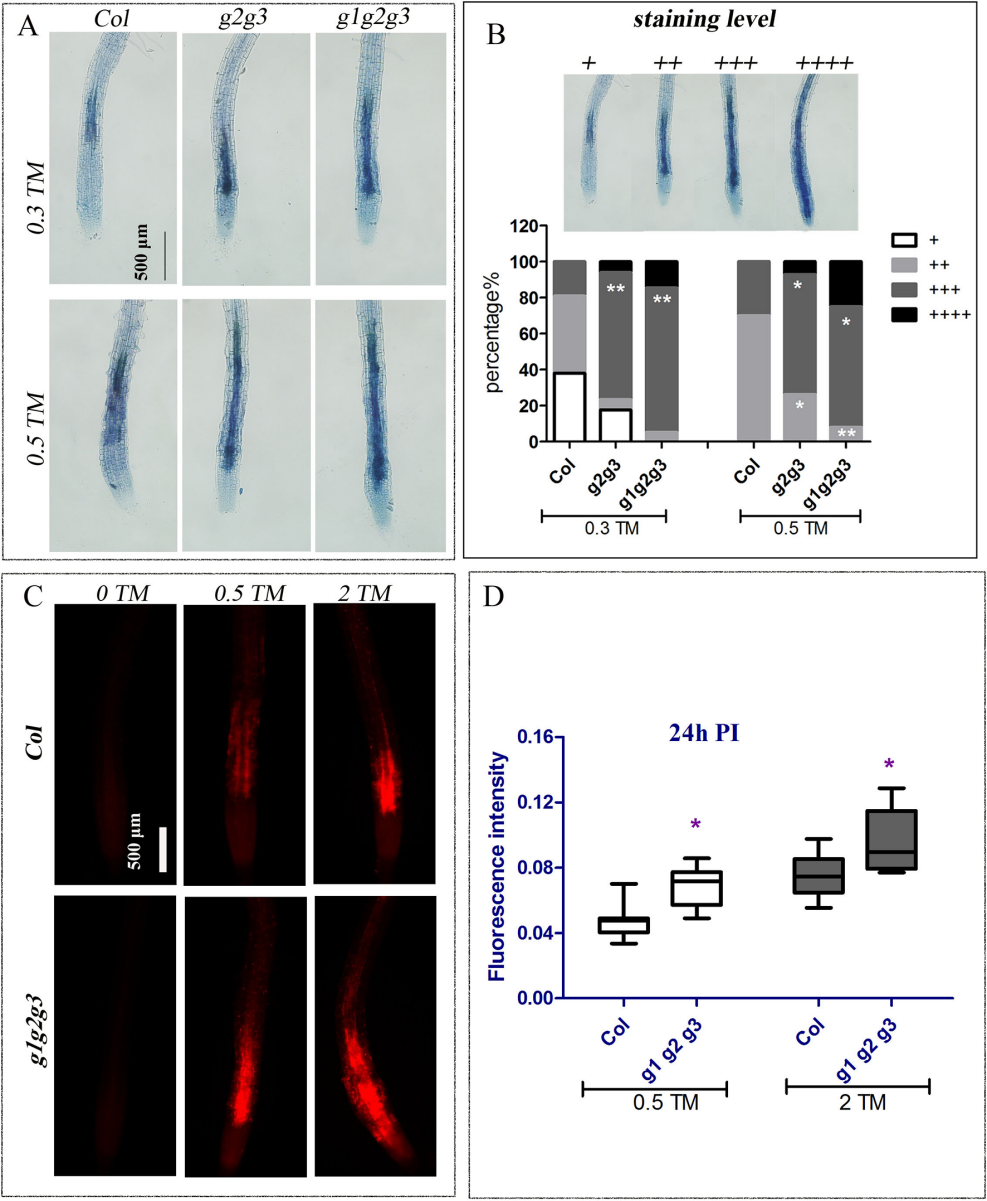
Mutation of *GAAP1*, *GAAP2* and *GAAP3* promoted ER stress-induced cell death on root. The seedlings of Col, *gaap2gaap3* and *gaap1gaap2gaap3* that were vertically cultured were transferred to a liquid medium containing different concentrations of TM (0, 0.30, 0.50, and 2.0 μg ml^−1^). **(A–B)** The root cells were assayed by trypan blue staining 48 h post treatment and representative images were shown **(A)**. The classification of staining level and the cell death severity were analyzed by quantifying the staining degree **(B)**. Data are from three biological replicates (± SE) and at least 20 samples for each plant line were used for each treatment (χ test, *p < 0.05, **p < 0.01). **(C–D)** The cell membrane permeability of root cells was assayed by PI staining 24 h post TM treatment **(C)**, and the fluorescence intensity by Image J program **(D)**. Significant differences compared with Col plants are indicated by asterisks (Student test, *p* < 0.05, n ≥ 30).

In addition, when seedlings were treated with 0.3 and 0.5 μg ml^−1^ TM for 48 h, more dead cells were found in the cotyledon and root of *gaap2gaap3* double mutants than in *gaap2* single mutants, whereas the least was found in root cells of *GAAP2* over-expression line (*GAAP2 in gaap2-1* #4) (**Supplementary Figures S4** and **S5**). The TM-induced cell viability of the triple mutant *gaap1gaap2gaap3* and double mutants *gaap2gaap3* were also evaluated with trypan blue and PI staining respectively. And the root cells of *gaap1gaap2gaap3* triple mutant showed the strongest and largest area of signals, followed by double mutant (**Figure 6**), indicating that the higher order of the mutations, the more severe the cell damage under TM. Under dark condition, the degree of yellow leaves *in vitro* in the presence or absence of TM was enhanced in the triple mutant (**Supplementary Figure S6**). These data indicated that mutations in *GAAP1*, *GAAP2*, and *GAAP3* played redundant roles in cell death resistance under ER stress.

**Figure 7:**
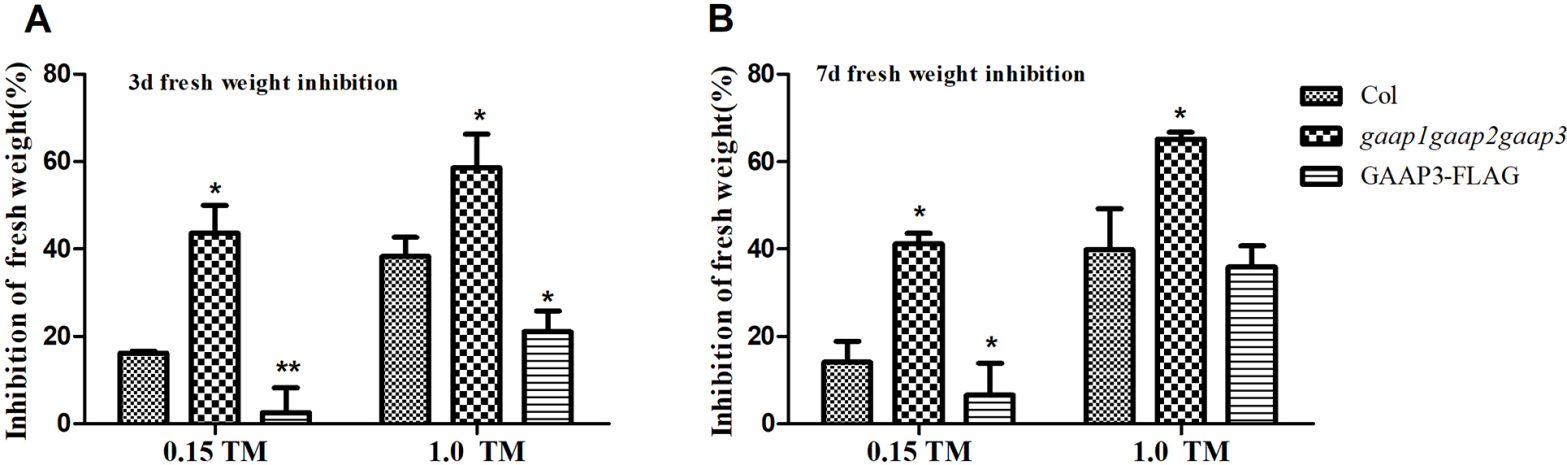
Mutation of *GAAP1*, *GAAP2* and *GAAP3* inhibited growth recovery after brief ER stress. (**A–B)** The 4-day-old seedlings that were infiltrated with 1/2 MS liquid salt containing 0, 0.15, and 1.0 μg ml^−1^ TM for 6 h were transferred to the 1/2 MS solid medium. The inhibition rates of the fresh weight of seedlings were determined after recovery for 3 and 7 days. Data are from three biological replicates (± SE), and at least 30 samples for each plant line were used for each treatment. Asterisks refer to significant differences from Col at the same time points and same conditions according to Tukey’s range (honest significant difference) test and two-way ANOVA (*p < 0.05, **p < 0.01).

*GAAP1* and *GAAP3* are conductive for plant growth during mild ER stress recovery (Guo et al., 2018). We further examined the growth of the triple mutant after a brief ER stress. The 4-day-old seedlings were infiltrated with 0, 0.15, and 1.0 μg ml^−1^ TM for 6 h and then transferred to the normal solid medium. Consistent with previous result, the decreased fresh seedling weight of the *GAAP3*-overexpression line was remarkably lower than that of the wild type, whereas the inhibition rate of *gaap1gaap2gaap3* seedling was higher (Guo et al., 2018) (**Figure 7**). These data showed that *GAAP1* to *GAAP3* played redundant roles in ER stress response.

### *GAAP1*, *GAAP2*, and *GAAP3* Affected the Mild ER Stress-Activated UPR

**Figure 8:**
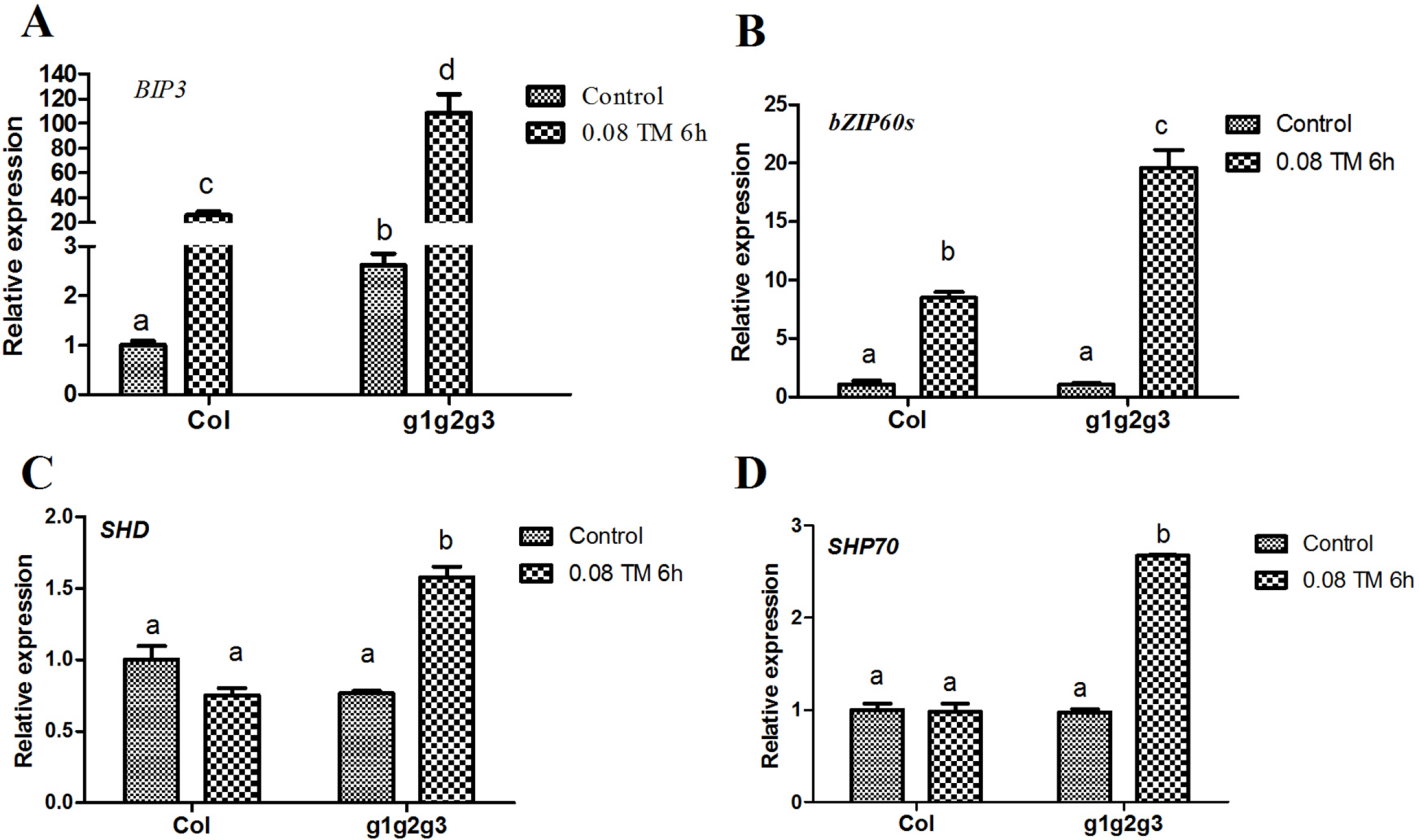
Mutations of *GAAP1*, *GAAP2* and *GAAP3* enhanced the up-regulation of mild ER stress-induced UPR gene. **(A–D)** The transcription levels of selected UPR marker genes in Col and *gaap1gaap2gaap3* seedlings treated with 80 ng ml^−1^ TM for 6 h were quantified by qRT–PCR. The value of each control of Col was set at 1. Error bars represent the standard deviation. Different letters indicate significant differences between different plants according to Tukey’s range (honest significant difference) test and two-way ANOVA (*p*< 0.05, n = 3).

Seedlings with mutations in *GAAP1*, *GAAP2* and *GAAP3* genes were more sensitive to ER stress than the wild type and double mutants. Under ER stress, UPR signaling pathways were initiated to alleviate stress (Walter and Ron, 2011; Deng et al., 2013). *GAAP1* and *GAAP3* slightly affect UPR under acute ER stress but play antagonistic roles in UPR gene induction at the beginning of mild ER stress (Guo et al., 2018). We further detected the transcription levels of the common target gene downstream of the UPR pathway in triple mutants treated with 80 ng ml^−1^ TM for 6 h, that is, short-term mild ER stress. The up-regulation of *BIP3*, *bZIP60s*, *CNXI*, *SHP70*, and *SHD* were all considerably higher in the *gaap1gaap2gaap3* than in the wild type (**Figure 8**). These data suggested that *GAAP1*, *GAAP2* and *GAAP3* played redundant negative roles in the mild stress-activated UPR protective response. Upon acute ER stress caused by 2.0 or 5.0 μg ml^−1^ TM for 6 h, the induction values of the markers in Col, *gaap2-1*, *gaap2gaap3*, and *gaap1gaap2gaap3* plants were similar (**Supplementary Figures S7** and **S8**), which suggested that *GAAP1 to GAAP3* mutations had little effect on the induction of UPR genes upon acute ER stress.

### Involvement of *GAAP1* to *GAAP3* in the Modulation of SA Signaling Inhibited Cell Death

**Figure 9:**
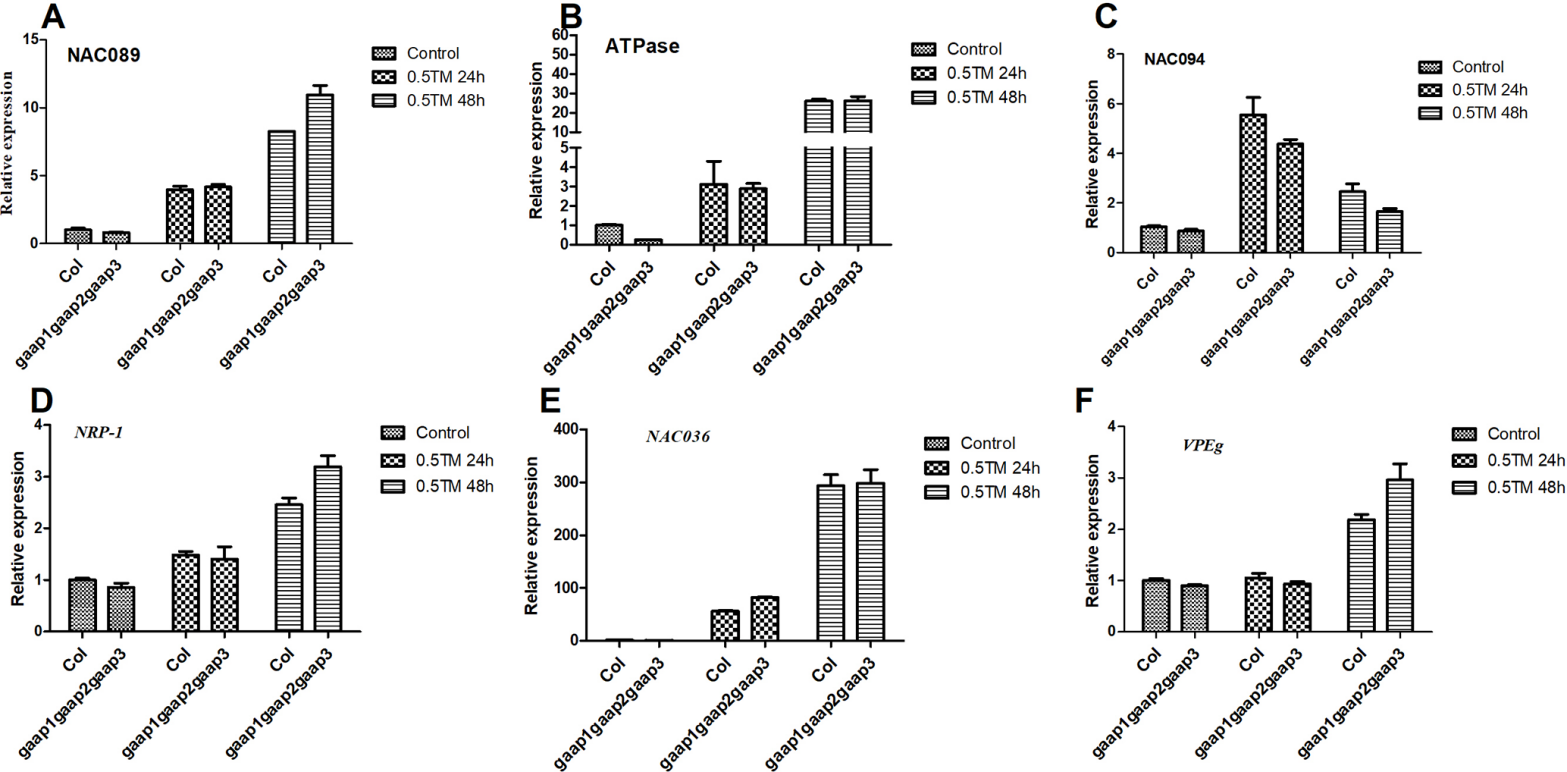
Upregulation of PCD genes of NAC089 pathway and DCD/NRP pathway by ER stress was not affected by mutations in *GAAP1*, *GAAP2* and *GAAP3*. **(A**–**F)** The seedlings of Col and *gaap1gaap2gaap3* treated with 0.5 μg ml^−1^ TM for 24 and 48 h. The expression levels of PCD genes in NAC089 pathway **(A**–**C)** and in DCD/NRP pathway **(D**–**F)** assayed by qRT-PCR. The value of each gene in Col under controlled condition was set at 1. Data were from three biological replicates (± SD). No significant differences compared with Col plants under the same condition were found (Student test, *p* < 0.05).

Under ER stress, IRE1 and bZIP28 can induce PCD by up-regulating NAC089, which promotes cell death by activating the expression of downstream PCD-related genes, such as *NAC094* and *ATPase* (Yang et al., 2014). We have shown that cell death began to appear when 0.3 or 0.5 μg ml^−1^ TM for 48 h. To clarify the effect of GAAPs on NAC089 pathway-mediated cell death, the gene expression levels of *NAC089* and its targets were analyzed at 24 and 48 h post TM treatment. The up-regulation of *NAC089*, *NAC094* and *ATPase* genes in *gaap1gaap2gaap3* was not remarkably different from that in wild type in response to TM treatment (**Figures 9A–C**). DCD/NRP also mediate cell death under ER stress (Reis et al., 2016). During ER stress, AtNRP1 activates downstream *AtNRP2* and *ANAC036*, thereby ultimately activating the vacuolar processing enzyme *VPEg* to induce PCD (Reis et al., 2016). The expressions of *AtNRP1* and *ANAC036* genes were up-regulated in both triple mutant and wild type under TM treatment, but no significant difference of upregulation was observed between them (**Figures 9D–F**). These data suggest that GAAPs inhibiting PCD might not be mediated by NAC089 and DCD/NRP pathways.

**Figure 10:**
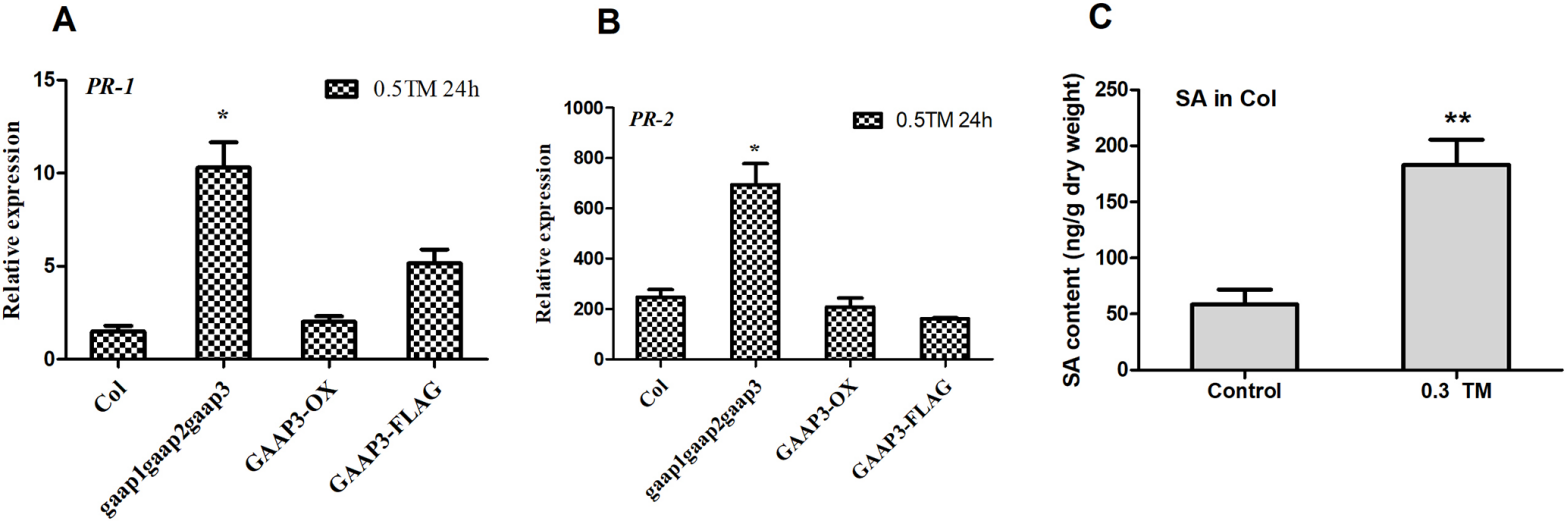
Mutations of *GAAP1*, *GAAP2*, and *GAAP3* enhanced the upregulation of the target genes downstream SA pathway under ER stress. **(A**–**B)** The seedlings of Col, *gaap1gaap2gaap3*, *GAAP3-OX*, and *GAAP3-FLAG* were treated with 0.5 μg ml^−1^ TM for 24 h, and the expression levels of SA pathway genes were quantified by qRT–PCR. The value of each gene in Col at 0 h was set at 1. **(C)** SA accumulation under ER stress. Total SA were extracted and measured from 5-week-old plants grown at 22°C or treated with 0.3 μg ml^−1^ TM for 48 h. Data are from four biological replicates (± SD). Data were from four biological replicates (± SD). Significant differences compared with Col plants were indicated by stars (Student test, *p < 0.05, ** *p* < 0.01)

In addition, the SA pathway also plays a key regulatory role in the resistance and promotion of PCD under stress (Yun and Chen, 2011; Gao et al., 2017). *PR-1* and *PR-2* are key target genes in the SA pathway. Quantitative detection showed that the expression levels of *PR-1* and *PR-2* in the triple mutant *gaap1gaap2gaap3* were considerably higher than those in the wild type and GAAP3 overexpressing lines at 24 h post TM treatment (**Figures 10A, B**). This finding suggests that the promotion of PCD by mutations in GAAP1 to GAAP3 under ER stress might be mediated by the SA pathway. To further determine whether SA content can be affected under ER stress, The total content of SA was measured from 5-week-old plants grown at 22°C or treated with 0.3 μg ml^−1^ TM for 48 h. And the result showed that SA level was upregulated by ER stress (**Figure 10C**).

## Discussion

*GAAP*s are broadly conserved cytoprotective proteins that are localized in the ER and Golgi apparatus membranes (Henke et al., 2011; Guo et al., 2018). *Arabidopsis GAAP1* and *GAAP3* played roles in resistance to ER stress and also in the delayed activation of UPR signaling pathway by interacting with UPR receptor IRE1 (Guo et al., 2018). The sensitivity of *gaap2* single, double or triple mutants with *gaap1* or *gaap3* to ER stress increased as the number of gene mutations increases assayed by plant or cell survival rate (**Figures 3**–**6** and **Supplementary Figure S4**), indicating that *GAAP1* to *GAAP3* played redundant roles in maintaining plant growth and survival under ER stress, at least by partially attenuating cell death. *GAAP1* and *GAAP3* genes were mainly expressed in the young seedlings and preferentially expressed in the reproductive organs, whereas *GAAP2* was strongly expressed in all parts throughout the entire development process (Guo et al., 2018)(**Figures 1** and **2**). The data suggested that different *GAAP*s might be involved in resistance to stress with tissue and developmental specificity. It’s worthy to note that *GAAP2* in *gaap2-1* showed only partial restoration under ER stress induced by DTT (**Supplementary Figure S1**). There are two possible reasons. One is that the gene driven by its own promoter without 3’-UTR region is not fully regulated. Another possible reason is that DTT, being a redox reagent, affects not only protein processing but also many other biological pathways dependent on the cellular redox state. And *GAAP2* might also participate in the regulation of other biological process with accurate expression levels.

IRE1A/B and bZIP28 are the sensors mediated two main pathways of UPR in plants (Liu et al., 2007; Mishiba et al., 2013). Unmitigated ER stress induces PCD in animals and plants (Lisbona et al., 2009; Yang et al., 2014). However, there is limited knowledge regarding how the activities of UPR sensors are regulated to ensure plant growth under different stress intensities. *Arabidopsis* BI-1 attenuates the pro-survival function of bZIP28, but it does not temper the ribonuclease activity of IRE1 in recovery roles from temporary ER stress (Ruberti et al., 2018). *GAAP1*/*GAAP3* function as the inhibitor of UPR dependent on IRE1 to reject the protective pathway at stress mitigation (Guo et al., 2018). Here, we showed that the upregulation of protective marker genes of both UPR pathways were considerably enhanced in triple mutant *gaap1gaap2gaap3* under mild ER stress (**Figure 8**) and no significantly different induction levels in most UPR genes were observed when plants were treated with high doses of TM, namely, 2–5 μg ml^−1^, for a few hours (**Supplementary Figures S7** and **S8**), indicating that *GAAP1* to *GAAP3* are fine modulator of UPR and might function as inhibitor in UPR protective response. The level of *GAAP*s might determine the sensitivity of cells to stress response to some extent. The mechanism needs further elucidation.

Under severe or persistent ER stress, cell death follows the induction of UPR (Watanabe and Lam, 2008; Ishikawa et al., 2011). The transcription factor NAC089 can be induced by IRE1 and bZIP28 to promote cell death by activating the expression of downstream PCD-related genes, such as *NAC094* and *ATPase* (Yang et al., 2014). DCD/NRP also mediates cell death under ER stress (Reis et al., 2016). The changes in gene expression levels in both pathways did not differ between wild type and triple mutant in response to TM treatment (**Figure 9**). However, the expression levels of *PR-1* and *PR-2*, which are key target genes in the SA pathway, were enhanced in the triple mutant after TM treatment (**Figure 10**). SA pathway also plays a key regulatory role in plant immunity and promotes PCD under stress (Yun and Chen, 2011; Pajerowska-Mukhtar et al., 2012; Gao et al., 2017). Being the production site of antimicrobial proteins and immune signaling components, ER functions as the central regulator in the execution of immune responses in plants (Pajerowska-Mukhtar et al., 2012). The UPR plays a fundamental role not only in abiotic stress responses but also in plant immunity (Moreno et al., 2012). Salicylic acid induces the expression of UPR genes, which in turn can induce the up-regulation of downstream genes of salicylic acid E (Watanabe and Lam, 2008; Moreno et al., 2012; Nagashima et al., 2014). Here we further showed that SA level was upregulated by ER stress (**Figure 10C**). These data suggested that the function of GAAP1 to GAAP3 against PCD might be achieved by reducing the upregulation of SA pathway under ER stress. AtBI-1 is involved in the inhibition of PCD in Arabidopsis by suppressing the ER-dependent reactive oxygen species (ROS) production or by regulating cell death-associated ER Ca^2+^ homeostasis (Watanabe and Lam, 2008; Watanabe and Lam, 2009; Ozgur et al., 2018). A close mutual induction relationship was observed between SA signaling pathway and ROS production. The detailed information regarding GAAP inhibiting PCD must be further studied.

In summary, GAAP1 to GAAP3 played redundant roles in maintaining plant growth and survival under ER stress, at least by partially resisting cell death. Upon mild ER stress, GAAP1 to GAAP3 negatively activated UPR pathway. ER stress also upregulated SA level in cells. GAAP1 to GAAP3 suppressed the upregulation of SA pathway against cell death under ER stress.

## Accession Numbers

The Arabidopsis Genome Initiative accession numbers for the proteins referred to in the paper are At4G14730 (GAAP1), AT3G63310 (GAAP2), At4G02690 (GAAP3), At3G10800 (bZIP28), At1g49240 (ACTIN8), At1G09080 (BIP3), At1G42990 (bZIP60), At5G24360 (IRE1B), At4G16660 (HSP70), At4g24190 (SHD), At5g61790 (CNX1), AT2G14610 (PR-1), AT3G57260 (PR-2), AT5G22290 (NAC089), AT5G39820 (NAC094), AT5G40010 (ATPase), AT2G03440 (AtNRP1), AT2G17040 (ANAC036), and AT4G32940 (GAMMA-VPE).

## Data Availability

All datasets generated for this study are included in the manuscript/supplementary files.

## Ethics Statement

No human studies are presented in this manuscript. No animal studies are presented in this manuscript. No potentially identifiable human images or data is presented in this study.

## Author Contributions

WW and MZ performed the assay about the plant sensitivity to ER stress. XinL performed the expression pattern and cell death pathway assay. WW, MZ and XT conducted the UPR assay. XT conducted the SA level detection. KG and ZW generated the mutants and transgenic plants. YZ performed gene expression assay. YS and WZ supervised the physiological experiments and participated in interpreting the morpho-physiological data. XL designed the experiment, supervised the study, analyzed the data and wrote the paper.

## Funding

This work was supported by the National Natural Science Foundation of China (No. 31670271).

## Conflict of Interest Statement

The authors declare that the research was conducted in the absence of any commercial or financial relationships that could be construed as a potential conflict of interest.

## Abbreviations

ER, endoplasmic reticulum; UPR, unfolded protein response, Bax, Bcl-2 associated X protein; PCD, programmed cell death; qRT-PCR, Quantitative real-time PCR; TM, tunicamycin; DTT, dithiothreitol; ROS, reactive oxygen species; BiFC, bimolecular fluorescence complementation; GUS, beta-Dglucuronidase; PI, Propidium iodide.
