# Supplementary material for: Arabidopsis GAAP1 to GAAP3 Play Redundant Role in Cell Death Inhibition by Suppressing the Upregulation of Salicylic Acid Pathway Under Endoplasmic Reticulum Stress
Source: Front Plant Sci. 2019 Aug 27;10:1032. doi: 10.3389/fpls.2019.01032 (PMC6719610; doi:10.3389/fpls.2019.01032)
Supplement: Supplementary file 1 [file Table_1.docx]

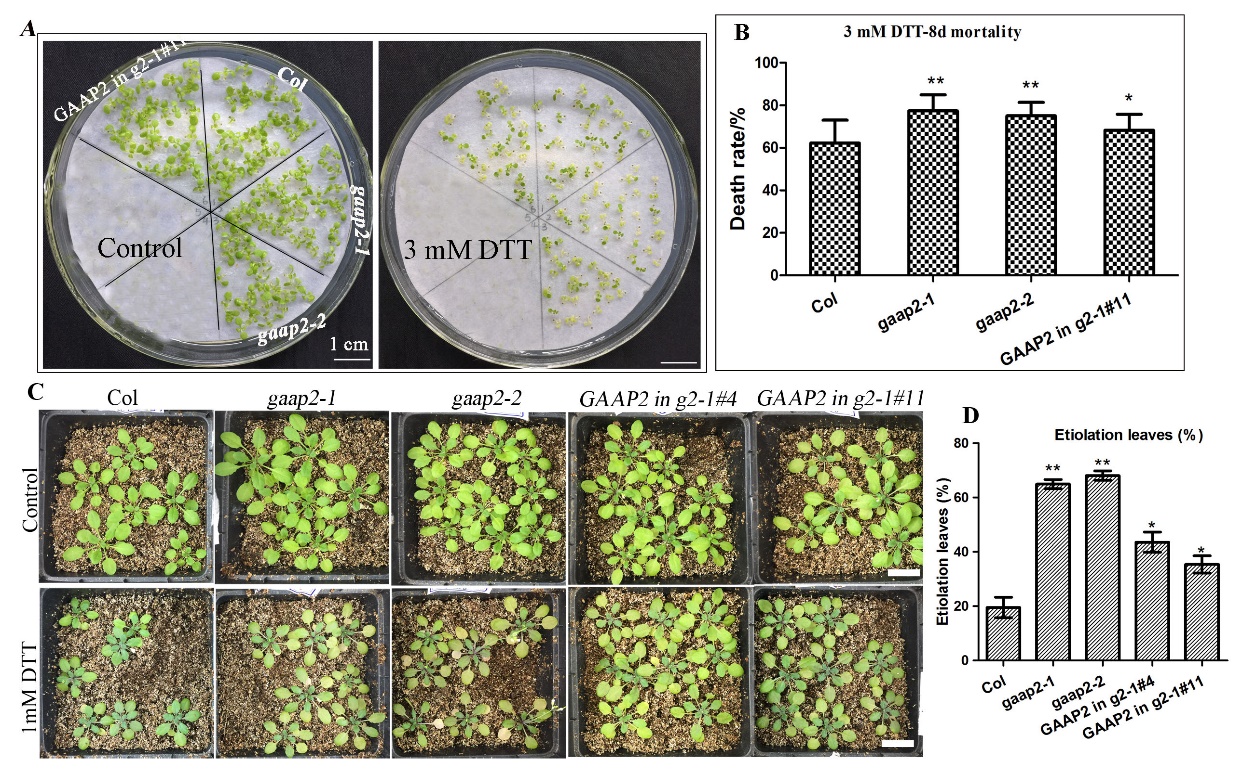


**Supplementary Fig.S1 GAAP2 enhanced the resistance of plants to ER stress under treatment with DTT.** (**A-B**) Col, *gaap2-1*, *gaap2-2*, and GAAP2 in *gaap2-1* seedlings were grown on 1/2 MS medium for 3 d and then transferred to a medium with 0–3 mM DTT for another 8 d (**A**) and the death rates of seedlings treated with DTT (**B**). Error bars depict a standard error of approximately 3–4 independent experiments. Significant differences compared with Col plants, as indicated by asterisks (*χ^2^* test, **p*<0.05, ** *P* < 0.01, n>80). (**C-D**) Growth (**C**) and leaf etiolation rates (**D**) of 15-day-old Col, *gaap2-1*, *gaap2-2*, *gaap2gaap3, GAAP2 in gaap2-1* #4 and #11, which were treated with 1 mmol L^-1^ DTT and recovered for 21 d. Bar = 2 cm, *n* = 9~16, mean ± SD, * *P* < 0.05, ** *P* < 0.01, *χ^2^* test.


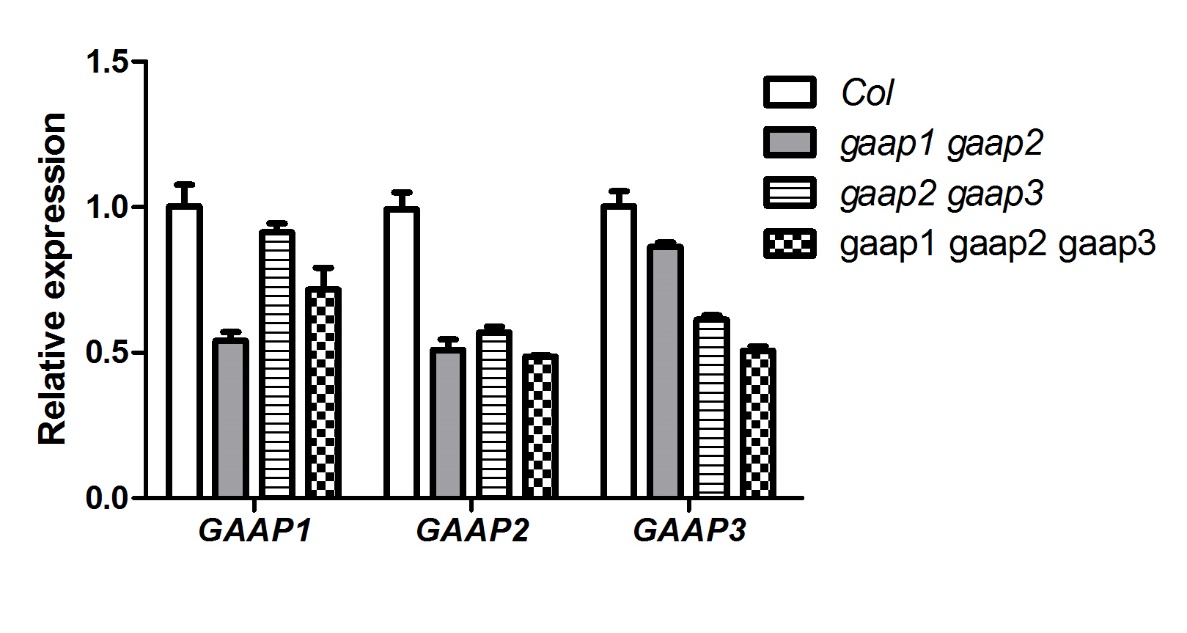


**Supplementary Fig.S2** The expression level of *GAAP1*, *GAAP2* and *GAAP3* in the 7-d old seedlings of *gaap1gaap2*, *gaap2gaap3* and *gaap1gaap2gaap3* assayed by qRT-PCR.


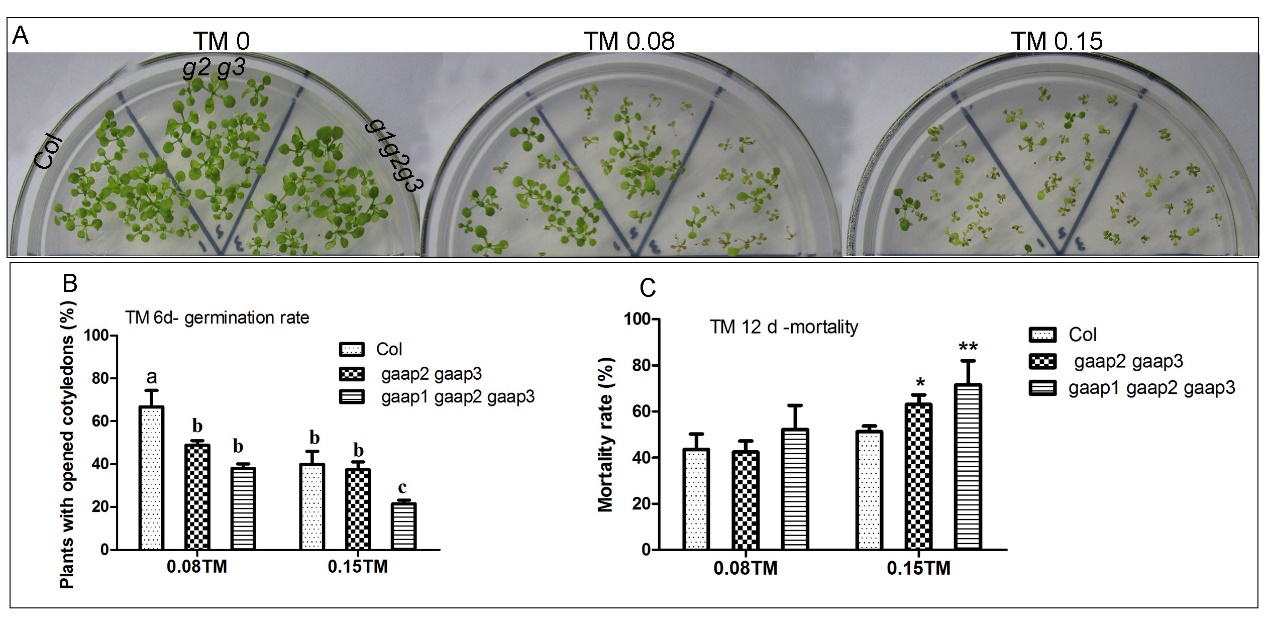


**Supplementary Fig.S3 The double and triple mutations in *GAAP1*, *GAAP2* and *GAAP3* enhanced plant sensitivity toward ER stress. (A)** Growth of Col, *gaap2gaap3*, and *gaap1gaap2gaap3* seedlings on 1/2 MS medium supplied with different concentrations ( 0, 0.08 and 0.15 μg mL^-1^ ) of TM for 12 days. **(B)** The percentage of seedlings with opened cotyledons which grew on 1/2MS medium with different concentrations of TM for 6 days. Error bars represent the standard error and different letters indicate significant differences between different plants subjected to χ test. **(C)** The percentage of death plants which grew on 1/2MS medium with different concentrations of TM for 12 days. Error bars depict standard error of 4 independent experiments. Significant differences compared with Col plants, as indicated by asterisks (*χ^2^* test, **p*<0.05, ** *P* < 0.01, n = 150).


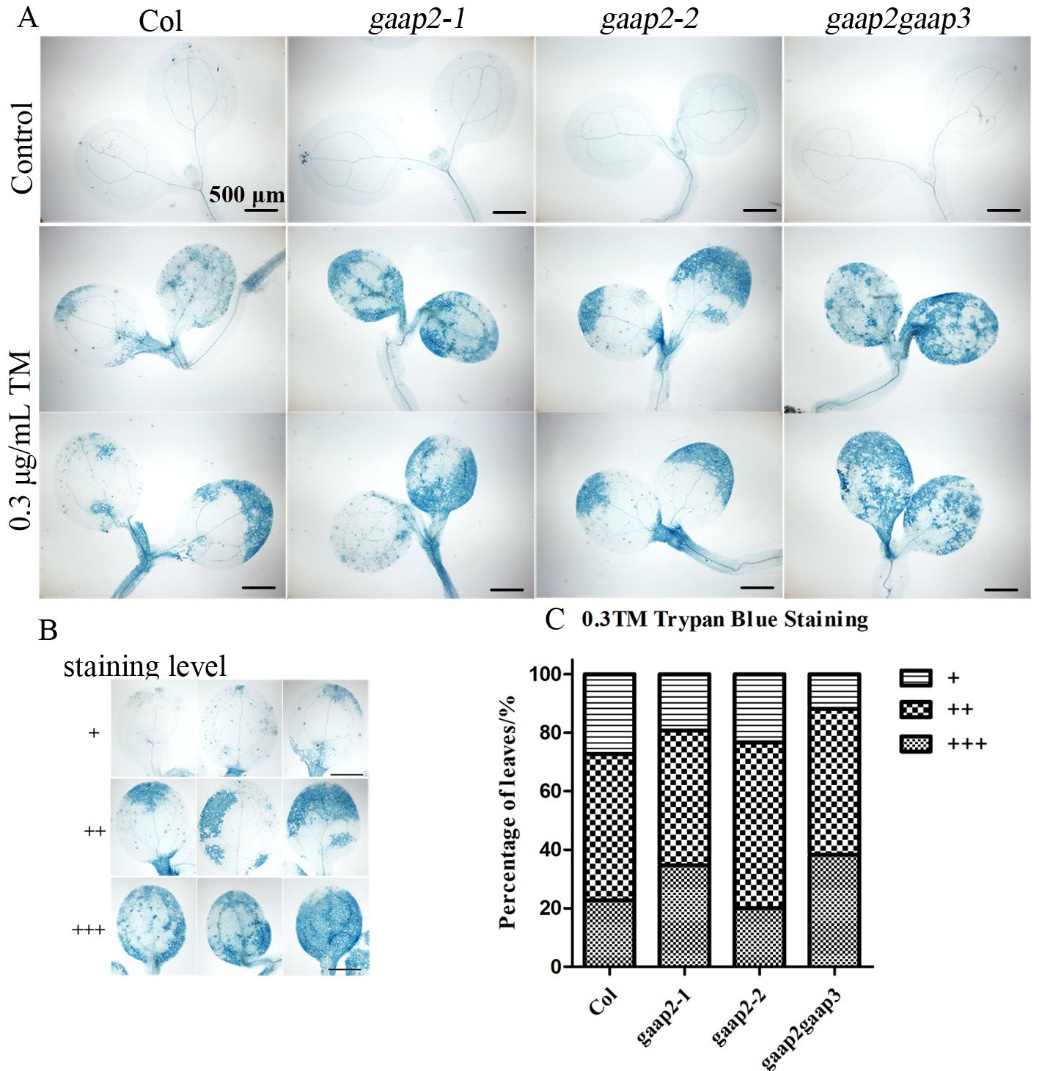


**Supplementary Fig.S4 GAAP2 and GAAP3** **inhibited the cell death induced by ER stress.** **(A)** The 3-day-old seedlings that were vertically cultured were incubated in 0.3 μg mL^-1^ TM for 48 h. Cotyledon cells of Col, *gaap2-1*, *gaap2-2* and *gaap2gaap3* were stained by trypan-blue. Bar = 500 μm. **(B-C)** The cell death severity was analyzed by quantifying the staining degree. Trypan blue staining intensity was classified in 3 levels, namely, faint, medium, and strong, and depicted as “+” “++” and “+++” respectively (B), and the percentage of each group was calculated (C). (n≧20).


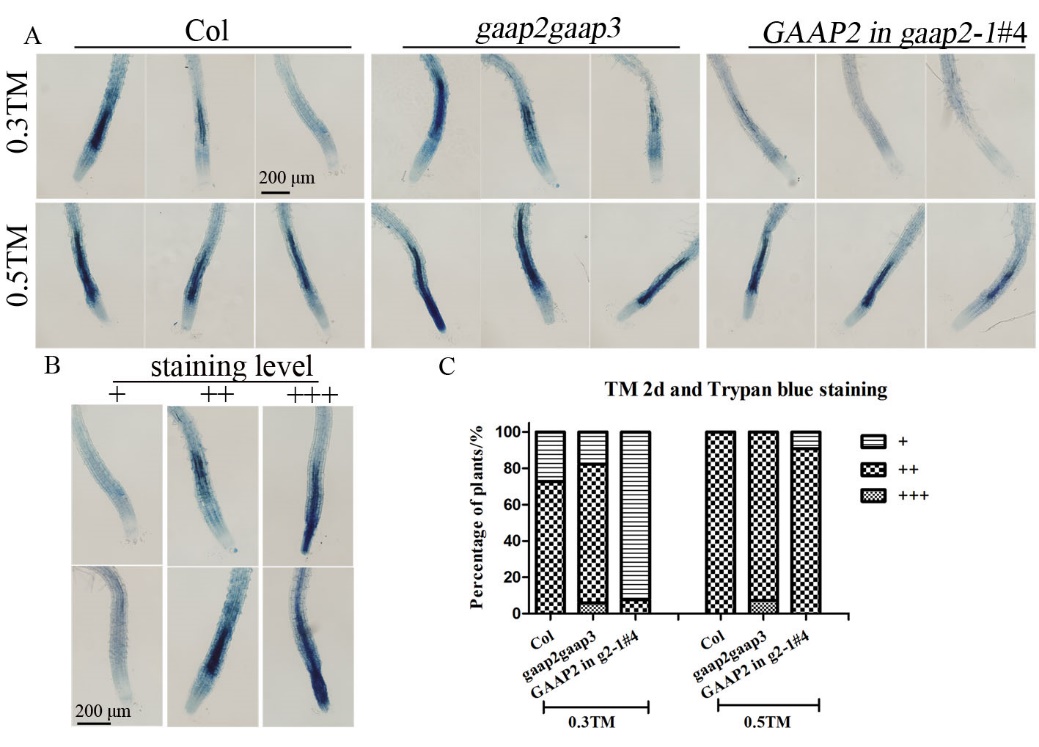


**Supplementary Fig.S5 GAAP2 and/ or GAAP3** **inhibited the cell death induced by ER stress.** **(A)** The 3-day-old seedlings that were vertically cultured were incubated in liquid medium containing different concentrations of TM for 48 h. Root cells of Col, *gaap2gaap3* and *GAAP2 in gaap2-1* #4 were stained by trypan-blue. **(B-C)** The cell death severity was analyzed by quantifying the staining degree. Trypan blue staining intensity was classified in 3 levels, namely, faint, medium, and strong, and depicted as “+”, “++” and “+++”, respectively (B), and the percentage of each group was calculated (C) (n≧20).


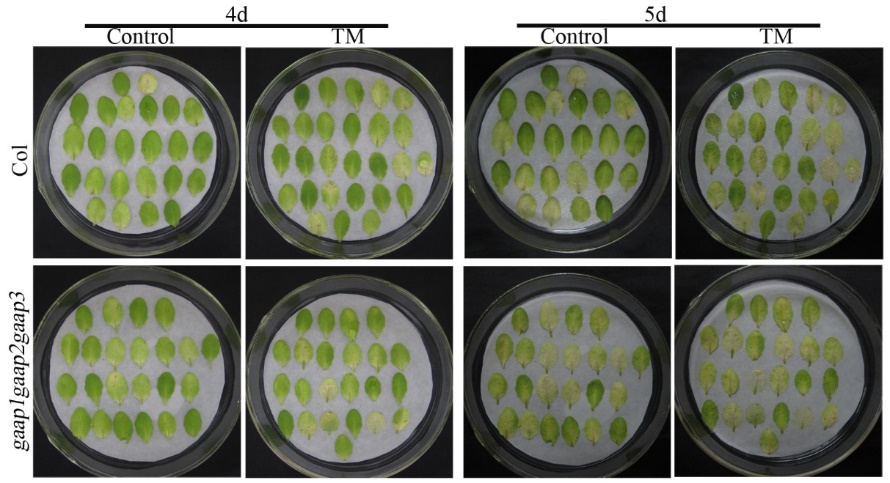


**Supplementary Fig.S6 The PCD induced by starvation was enhanced in *gaap1gaap2gaap3* mutant.** The leaves were taken from 30– day old of Col and *gaap1gaap2gaap3* which were grew in soil and put on the wet filter paper containing 0 and 0.15μg mL^-1^ TM under dark for 4 d and 5d.


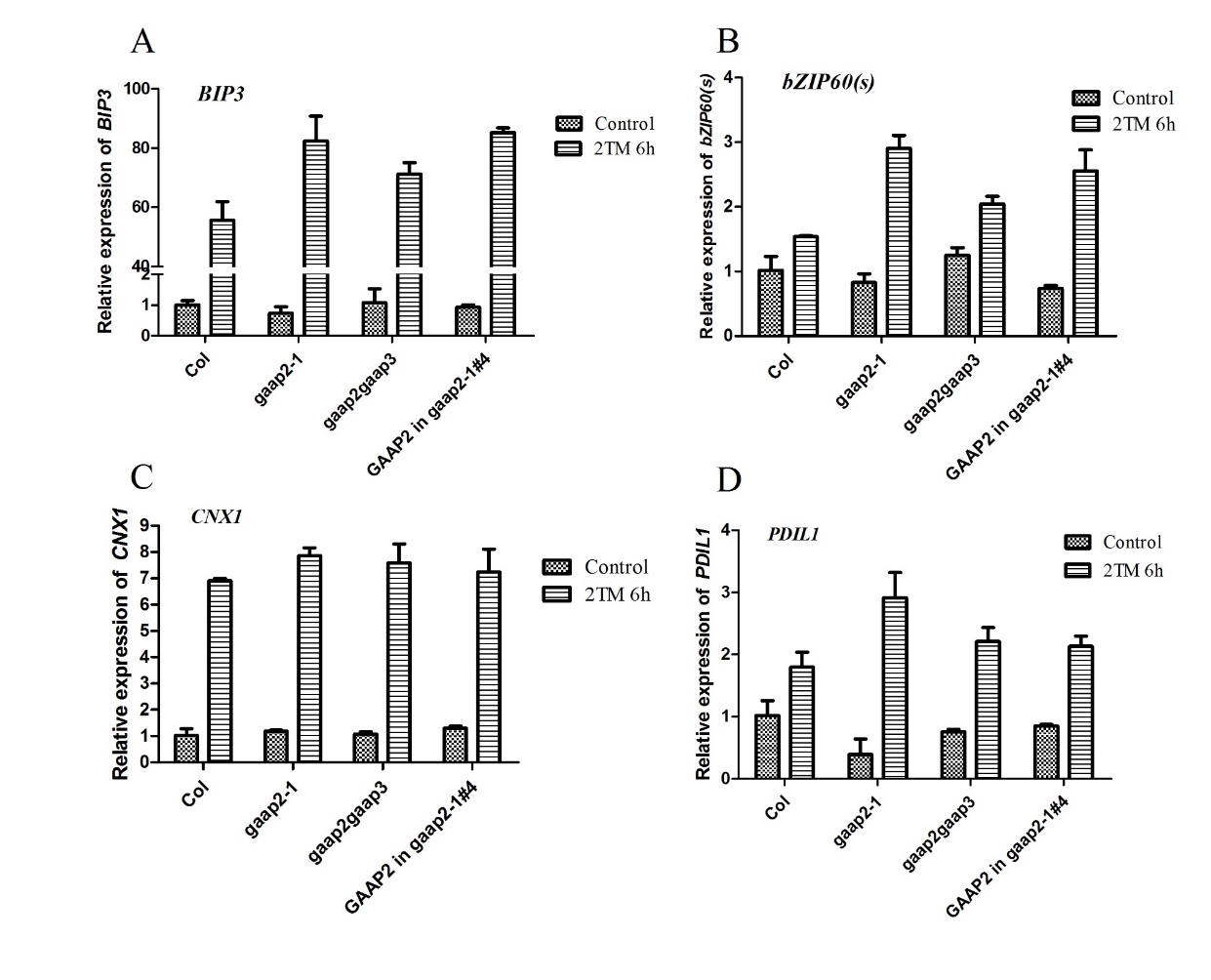


**Supplementary Fig.S7** ***GAAP2* mutation or overexpressing had little effect on the induction of UPR genes upon acute ER stress.** Total RNA was isolated from 7 days–old seedlings of Col, *gaap1-1* and 35S::GAAP1-OX#4 were treated with 2.0 μg mL^-1^ Tunicamycin or 0.1% DMSO (control) for 6 h. The transcript levels of selected ER marker genes were quantified by qRT–PCR. Gene expression was normalized to ACTIN8 and the value of each control of WT (Col) was set at 1. Data are from 3 to 3 biological replicates (±SD). No statistically significance between different plant lines according to Tukey’s range (honestly significant difference) test and two-way analysis of variance (*p*< 0.05).


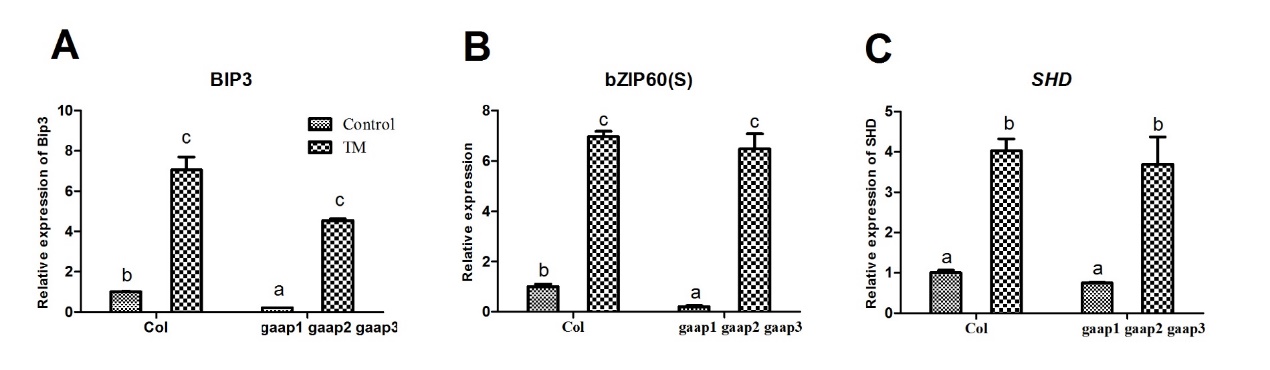


**Supplementary Fig.S8** ***GAAP1 to GAAP3* mutations had little effect on the induction of UPR genes upon acute ER stress.** Total RNA was isolated from 7 days–old seedlings of Col, *gaap1-1* and 35S::GAAP1-OX#4 were treated with 5.0 μg mL^-1^ Tunicamycin or 0.1% DMSO (control) for 6 h. The transcript levels of selected ER marker genes were quantified by qRT–PCR. Gene expression was normalized to ACTIN8 and the value of each control of WT (Col) was set at 1. Data are from 3 to 3 biological replicates (±SD). Statistically significance was analyzed according to Tukey’s range (honestly significant difference) test and two-way analysis of variance (*p*< 0.05).

**Supplementary** Table S1. Primers used.

| gene/mutant/constructs name | T-DNA lines/AGI number | Purpose | Primer name | Primer sequences (5' to 3') |
| --- | --- | --- | --- | --- |
| gaap2-1 | CS814747 | Genotyping | LP | TGATCGGTACGGTACTCCTTG |
|  |  |  | *RP* | CTGGCAGAGATCTTTTCAACG |
| gaap2-2 | SALK_052507C | Genotyping | LP1 | GGAGGGACCTTCACAAAGAAG |
|  |  |  | RP1 | AATCTGTGATTTTGACCGCTG |
| GAAP2 | AT3G63310 | RT-PCR | AT3G63310 F2 | agatccAAGATGTGGAATCAGAAGCATGA |
|  |  |  | AT3G63310 R2 | gaattcTCTGAAAGAGACAAGACTGCCTAA |
| PGAAP2::GUS | AT3G63310 | GUS assay | AT3G63310 PF1 | AT3G63310 PF1 |
|  |  |  | AT3G63310 PR1 | AT3G63310 PR1 |
| PGAAP2::GAAP2 | AT3G63310 | Full gene driven by its own promoter in mutant | AT3G63310 PF1 | AT3G63310 PF1 |
|  |  |  | AT3G63310 R1 | AT3G63310 R1 |
| PR2 | AT3G57260 | qRT-PCR | PR2 qFW | AAGGAGCTTAGCCTCACCAC |
|  |  |  | PR2 qRV | CACAACGTCCGATGGACTTG |
| PR1 | AT2G14610 | qPCR for PR-1 | PR1 qFW | CCTGGGGTAGCGGTGACTT |
|  |  |  | PR1 qRV | CGTGTTCGCAGCGTAGTTGT |
| NAC089 | [AT5G22290](https://www.arabidopsis.org/servlets/TairObject?id=134971&type=locus) | qRT-PCR | NAC089 qPCRF1 | ACTGGGAAAGAGCGTGATGT |
|  |  |  | NAC089 qPCRR1 | TGGTGCCTTCTGACTTGTAC |
| NAC094 | AT5G39820 | qRT-PCR | NAC094 qPCR F1 | TCCAACGATTCCTGACAACA |
|  |  |  | NAC094 qPCR R1 | TCGTTGACTGGAGAAGGAAT |
| ATPase | AT5G40010 | qRT-PCR | ATPaseqPCRF1 | GACTAATACGGGCTCTGCTC |
|  |  |  | ATPaseqPCRR1 | CATAGACATCGCTTCGCTTG |
| AtNRP1 | AT2G03440 | qRT-PCR | qRT-AtNRP1 F | CAAACGCCAGCTTTTCGGAT TG |
|  |  |  | qRT-AtNRP1 R | TGAGCACGCTCTTCTTGCTTT CA |
| ANAC036 | AT2G17040 | qRT-PCR | qRT-ANAC036 F | TCCTCTTTCGTCTTCCGAGA |
|  |  |  | qRT-ANAC036 R | TGCATTTGGATCTTGTTTGC |
| GAMMA-VPE | AT4G32940 | qRT-PCR | qRT-VPEg F | TGCTGGGCAACCTCTAGTC |
|  |  |  | qRT- VPEg R | GTACTGAGACAGCGATCCA |
| BiP3 | At1g09080 | qRT-PCR | BiP3 F1 | CGAAACGTCTGATTGGAAGAA |
|  |  |  | BiP3 R1 | GGCTTCCCATCTTTGTTCAC |
| ACT8 | AT1G49240 | qRT-PCR | Act8 F1 | TCAGCACTTTCCAGCAGATG |
|  |  | qRT-PCR | Act8 R1 | ATGCCTGGACCTGCTTCAT |
| bZIP60 | AT1G42990 | qRT-PCR | bZIP60 F4 | GAAGGAGACGATGATGCTGTGGCT |
|  |  | qRT-PCR | bZIP60U B1 | GCAGGGATTCCAACAAGAGCACAG |
|  |  | qRT-PCR | bZIP60S B2 | AGCAGGGAACCCAACAGCAGACT |
| PDIL1 | At1g21750 | qRT-PCR | qPDIL1F | CTCGTGAAGCTGAGGGTATTG |
|  |  | qRT-PCR | qPDIL1R | TGTGCGAAATCTAACTCAGAG |
| SHD | AT4G24190 | qRT-PCR | qSHD F | GAAGGAAGCATTCAAGGAGCTA |
|  |  | qRT-PCR | qSHD R | TCTTTGATGATAGGGTGTCGTG |
| HSP70 | AT4G16660 | qRT-PCR | qHSP70 F | GGACTTGGAGGACAGAGTATGG |
|  |  | qRT-PCR | qHSP70 R | GGGTAATTGTGCTCCTGAAGTC |
| CNX1 | At5g61790 | qRT-PCR | CNX1 qFW | TTCTTCTCGCTCTTCCTCAAGC |
|  |  | qRT-PCR | CNX1 qRV | GCGGTTTCTTCCTTCTTCTCC |
